# Mental health risk in human services work across Europe: the predictive role of employment in various sectors

**DOI:** 10.3389/fpubh.2024.1407998

**Published:** 2025-01-13

**Authors:** Ágnes Győri, Éva Perpék, Szilvia Ádám

**Affiliations:** ^1^HUN-REN Centre for Social Sciences, Institute for Sociology, Budapest, Hungary; ^2^HUN-REN Centre for Social Sciences, Child Opportunities Research Group, Budapest, Hungary; ^3^Faculty of Health and Public Services, Semmelweis University, Health Services Management Training Centre, Budapest, Hungary

**Keywords:** EU Labor Force Survey, international comparison, mental health risks at work, social care, healthcare, education, human services

## Abstract

**Background:**

Human services occupations are highly exposed to mental health risks, thus psychosocial risk management is critical to assure healthy and safe working conditions, promote mental health and commitment, and prevent fluctuation of employees. However, still little is known about prominent psychosocial risk factors in various human services work.

**Objectives:**

To identify prominent psychosocial risk factors of mental health in human services occupations and to explore their individual and organizational correlates in 19 European countries.

**Methods:**

Cross-sectional survey using data from the European Union's Labor Force Survey among 379,759 active employees in 19 European countries. First, a descriptive analysis was carried out to establish the prevalence of mental health risk factors. Then sociodemographic correlates of occupational mental health risk factors were assessed by means of Pearson's chi-squared test. Finally, correlations were explored between perceived psychosocial risk factors and human vs. non-human services occupations, as well as contextual variables by applying multilevel logistic and multinomial regression analyses.

**Results:**

The prevalence of mental health risk was 45.1%. Work overload (19.9%), dealing with difficult clients (10.2%), and job insecurity (5.8%) were the most prevalent mental health risk factors among European employees. We identified significant differences in the prevalence of mental health risks and specific mental health risk factors among employees according to sex, age, and educational attainment. The prevalence of mental health risks was significantly higher among women (47.0%, man: 43.3%), workers aged 35–50 years (47.5%, >50: 44.4%, <35: 42.3%), and those with the higher level of education (51.9%, secondary with diploma: 42.6%, elementary: 36.2%). Employees working in healthcare in Northern Europe were most likely to be exposed to mental health risks (AME = 0.717). Working in healthcare in Northern Europe was the strongest predictor of reporting work overload (AME = 0.381). Working in social care in Central and Eastern Europe was the strongest predictor of reporting dealing with difficult clients (AME = 0.303) as the most prevalent mental health risk factor.

**Conclusion:**

Understanding the impact of employment in specific human services occupations on mental health and its specific occupational stressors are vital to improve mental health and safety at work and maintain high quality services.

## 1 Introduction

Human resources are the most important resource of society and economy, therefore health and wellbeing of workforce is of paramount importance. Today, work-related health impairment is increasingly mental than physical in nature. However, mental health is crucial for full-fledged work and individual wellbeing from one side, and for social cohesion and economy from the other. And vice versa: a decent work is crucial for mental health (1, 2, 52). Inadequate and/or harmful working environment—such as job insecurity, unbalanced workloads, low autonomy—endanger mental health. In 2019, globally 15% of working-age population were estimated to live with mental disorder. As a result, an estimated 12 billion working days are lost every year to anxiety and depression leading to US$ 1 trillion of lost productivity per year. Healthy and safe working conditions promote mental health, wellbeing, performance and commitment of employees and prevents fluctuation. Therefore, strategies to address mental health at work is inevitable. Strategies may include prevention and psychosocial risk management at work (e.g. flexible work schedule, anti-violence measures), protection and promotion (e.g. managerial and worker trainings) and support for those with lived experiences (3).

Preserving and protecting mental health at work is one of the priorities of the European Union (EU). Since 1989, the EU has been promoting improvements in occupational safety and health through legal instruments (Council Directive 89/391/EEC of 12 June 1989). Since 2014, the Framework Agreement on Work-Related Stress has provided European employers and workers with a framework for identifying, preventing or managing work-related stress. The agreement highlights that stress is not an individual, but a collective responsibility. On the other hand, the EU Strategic Framework on Health and Safety at Work 2021–2027 identifies the following psychosocial risks: excessive workloads, conflicting demands and lack of role clarity, lack of influence and lack of involvement in making decisions, poorly managed organizational change, job insecurity, ineffective communication, lack of support from management or colleagues, psychological and sexual harassment, and third party violence. In the wake of previous traditions, the latest European position is a comprehensive approach to mental health (52), and so is the global one (1).

In the EU, a comprehensive assessment of the relationship between work and physical and mental health has been conducted in all sectors since 1990. According to the data, almost one in four Europeans surveyed after the turn of the millennium was affected by work-related stress and this was the second most common occupational health problem (4, 5). On the other hand, job satisfaction has improved (80%) in Europe, supposedly leading to better health (6). It has also been shown that workplaces carried fewer health risks (7). However, insecurity of job and workplace, emotional demands, ethical conflicts and high requirements have emerged as new psychosocial risk factors. Lack of autonomy and work intensity have proven to be persistent problems in Europe. Meanwhile, a high level of work creativity was seen after 2010, which had a positive impact on mental health (8). These results obtained in specific nation-states are, of course, strongly influenced by the relevant labor market regulations and the health care system concerned (9).

Overall, the 2015 wave of the European Working Conditions Survey reported better mental health among workers (10). At the same time, according to some national data, overwork remained a significant problem. For instance, the number of employees working more than 48 h a week increased by 15% since 2010 in the United Kingdom (11). This work beyond working hours greatly reduces the time to recover and recuperate, which increases the risk of mental and other health problems.

Right before the outbreak of the coronavirus, the main challenges of the European working life were as follows: increasing psychosocial risks linked to emotional demands and exposure to hostile social behavior (especially in women-dominated sectors); increasing work intensity in some sectors (particularly in service and sales occupations); blurred work and non-work boundaries due to flexibility in the place and time of work (12). The latter problem even intensified after the outbreak of COVID-19. The pandemic caused unprecedented and far-reaching changes in working life including digitalisation, work-life balance, engagement and trust. During the pandemic, 30% of workers were marked with strained jobs in which the job demands were higher than the job recourses. Improved work quality combined with high anxiety rates and physical and mental exhaustion affecting more than one in 10 employees were also observed. In terms of sociodemographics, during COVID-19, men's mental wellbeing was better than that of women and, unexpectedly, mental health was better among those with primary education than that of tertiary education. Work-related stress levels are still high today, albeit lower than the 28% rate measured in the last 10 years (12).

Within and in addition to research covering the entire national economy, numerous studies have drawn attention to the mental health risks of the human professions. It has been proven that clients' emotional needs, especially in case of professions providing care and support, increase the risk of mental illness (10). Results by Johnson et al. (13) also show that, teachers and social workers, alongside paramedics, call center clerks, police officers and prison officers, are among the six professions characterized by below-average psychological and physical wellbeing and job satisfaction.

The three sectors with the highest health risks in Europe during the pandemic were health, agriculture and education. Thus, two out of three sectors belong to the human services sphere (12). A study of teachers in Italy found that half of the professionals surveyed exceeded the reference cut-off figure for depression and one-tenth for anxiety (14). According to the results of Bauer et al. (15) in Germany, nearly a third (30%) of teachers struggle with mental health problems.

The healthcare sector received a lot of attention worldwide before, during, and after the pandemic due to the significant physical and emotional strain on the professionals involved in the field (16–18). For instance, the research by Teles et al. (19) in Brazil found that at least one in seven primary healthcare workers has a poor quality of life. During the pandemic, seminal research studied the mental health of health and social workers from a comparative perspective. Jordan et al. (20) found no meaningful difference, while others showed higher mental burden among those working in the social sector (21, 22).

### 1.1 Study aims

A significant body of evidence in the literature clearly reveals that human services professions involving intense emotional work are associated with higher levels of distress and a higher risk of burnout (3, 23–26, 53). However, limited information is available on the prevalence of psychosocial mental health risk factors among employees in various human services occupations. To address this, we aim to identify prominent psychosocial risk factors of mental health and explore their prevalence and associations with specific human services professions such as healthcare, social services, and education as well as other sociodemographic and organizational factors among employees across Europe, i.e., Northern, Southern, Western and Eastern Europe.

## 2 Materials and methods

### 2.1 Data source

Our analyses were conducted using data from the international database of the 2020 supplementary data collection of the European Union's Labor Force Survey (EU LFS). This survey constitutes the highest harmonized data collection of social statistics in the EU (27). Since the turn of the millennium, an additional survey, a so-called *ad hoc* module, has been appended to the basic questionnaire of the labor force survey in the second quarter of the year. For the purpose of our analysis, we used the database of this *ad hoc* module, as this survey assessed the risks to mental health at work, which were key to our research, in more detail compared to previous years.

The dataset includes information on 457,430 respondents from 30 national random samples collected through the telephone using computer-assisted questionnaires, or in some cases by means of face-to-face interviews. Countries not having sufficient data quality in terms of two key variables of the analysis (type of occupation and reporting exposure to mental health risk) were excluded from the analysis, thus 19 countries are included in our study. After deleting cases with missing values on our variables of interest,^1^ and dropping individuals being older than 65 years of age or inactive as labor force (students, retired people, etc.), our analysis sample consists of 379,759 individuals. Since relevant literature (28) suggests that the types of welfare system influence the prevalence of psychosocial risks at work, we identified four groups of countries, taking into account the types of welfare systems. To distinguish welfare states, we rely on the typology of Soede et al. (29), who identified five distinct groups of welfare: Nordic, Continental, Anglo-Saxon, Mediterranean and Eastern European welfare states. As the UK, even though it belongs to the Anglo-Saxon countries, were not involved in the 2020 data collection of LFS due to the fact that it is not a member state of the EU any more, and Ireland was also excluded from the analysis due to the considerable lack of key variables, the typology we applied distinguishes only four groups of welfare states and not five. The four groups are the following in this study: (1) Western Europe: Austria, Belgium, Switzerland, Germany, France and Luxembourg, (2) Northern Europe: Denmark, Finland, Norway and Sweden (3) Central and Eastern Europe: Czech Republic, Estonia, Hungary, Lithuania, Latvia and Romania, and (4) Southern Europe: Greece, Italy and Spain.

### 2.2 Measures

#### 2.2.1 Dependent variables

To identify psychosocial risk factors of mental health at work, we selected one question from the survey inquiring about the most important factor adversely impacting mental wellbeing among the employees. The answer categories to the question were as follows: (1) no significant risk factor for mental wellbeing, (2) mainly severe time pressure or overload of work, (3) mainly violence or threat of violence, (4) mainly harassment or bullying, (5) mainly poor communication or cooperation within the organization, (6) mainly dealing with difficult customers, patients, pupils, etc., (7) mainly job insecurity, (8) mainly lack of autonomy, or lack of influence over the workplace or work processes, (9) mainly another significant risk factor for mental wellbeing.

Based on the nine response options, we ordered the data into three variables for the analyses. (1) The first was a dummy variable with a value of 0 indicating that the respondent was not exposed to any occupational psychosocial health risk, and 1 indicating that the respondent was exposed to one of the risk factors assessed. (2) The second variable was a revised set of eight psychosocial risk factors by merging the categories “mainly to violence or threat of violence” and “mainly to harassment or bullying” due to similar content. (3) As the number of items was low, we further reduced the response categories to five options as follows: (1) not exposed to mental health risk factors, (2) the main risk factors are overtime, time pressure, (3) the main risk factor is management of difficulties concerning clients, patients and children, (4) the main risk factor: job insecurity, (5) other main risk factors, including harassment and violence in the workplace, lack of autonomy at work, inadequate communication and cooperation within the organization, and other factors.

#### 2.2.2 Independent variable

To categorize the respondents according to the type of their occupation, we utilized the ISCO-08 (International Standard Classification of Occupations) occupational codes available in the LFS database. According to our research goal, we created five categories: (1) social care occupations, (2) healthcare occupations, (3) educational occupations, (4) other human services occupations that do not directly serve the personal health or social wellbeing of individual people, but their activities apply to the society as a whole or smaller or larger communities and client groups, and (5) non-human services occupations (see the Supplementary Appendix S1 for a detailed description of the occupations in each occupational category).

#### 2.2.3 Contextual variables

In order to explore correlates of mental health risk factors, we developed contextual variables that were included as controls, which have been associated with various psychosocial risks at work. One of our contextual variables was the existence of national-level legislation on psychosocial risks and/or work-related stress. According to previous research by Jain et al. (30), we created a discrete variable where 0 indicated that a country did not have specific legislation on work-related stress and 1 indicated that a country did have specific legislation (direct or indirect) on work-related stress. As of 31 December 2020, 63.2% (12 countries) of the countries selected for the analyses had specific national legislation on psychosocial risks and/or work-related stress, while 36.8% (seven countries) had no specific national legislation. We also used an additional national-level variable, the so-called “Psychosocial Risk Management Index” (PRM Index), developed by van Stolk et al. (31) and Lunau et al. (32) using the database of the European Survey of Enterprises on New and Emerging Risks (ESENER) conducted by EU-OSHA (European Agency for Occupational Safety and Health) in 2019. The PRM Index was created based on six variables each of which assessing the psychosocial risk management practices of the employer/organizations as judged by managers. We used these questions: (1) whether employees had been informed about psychosocial risks and their effect on health and safety, (2) whether the organization had a procedure to deal with work-related stress, (3) whether the organization used health and safety services (e.g., use a psychologist), (4) whether training(s) had been provided in the last 3 years to employees dealing with psychosocial risks, (5) whether workers were informed of whom to turn to in case of work-related psychosocial problems, (6) whether they used support from external sources on how to deal with psychosocial risks at work. The average scores calculated at the organizational level were summed and averaged at the national level. The resulting values of the composite PRM index ranged from 0 to 6, where higher values indicated a higher degree of psychosocial risk management at the company or organizational level in the country.

#### 2.2.4 Control variables

We also collected sociodemographic variables and working conditions at the individual level: sex, age (categorical variables: under 35, 35–50 years, over 50 years), educational attainment [categorical variables: primary (ISCED 0–2), secondary (ISCED 3–4), higher (ISCED 5–6) education], atypical employment (dummy variable: whether the interviewee worked on a fixed-term contract), atypical work patterns (dummy variable: whether the interviewee worked shifts and/or weekend and/or evening/night schedule) and work experience (continuous variable: number of years at current employment).

### 2.3 Statistical analysis

Statistical analyses were performed using STATA 16.0 (Release 16. College Station, TX: StataCorp LLC) software. First, a descriptive analysis was carried out to establish frequencies of mental health risk factors in the sample. Sociodemographic correlates of occupational mental health risk factors were assessed by means of univariate analysis using Pearson's chi-squared (χ^2^) test. The level of significance was set at *p* < 0.05. Subsequently, we explored correlations between perceived psychosocial risk factors of mental health and the type of occupations (human vs. non-human services) as well as contextual variables by applying multilevel logistic and multinomial regression analyses with random intercepts at the country level. We examined a blank model first in each case, without explanatory variables, in order to see whether there is a complex variance behind the structure of the dependent variables. The models provided a better match when the clustering of data was taken into account, suggesting that the impact of the individual and national levels affects the output variable independently. Multicollinearity among explanatory variables was checked using variance inflation factor (VIF).

## 3 Results

### 3.1 Background characteristics of the sample

The characteristics of the sample are depicted in Table 1. The dataset contains information on 379,759 respondents from 19 countries, with the highest number of respondents in Italy (*N* = 42,760) and the smallest number in Latvia (*N* = 3,969). Slightly more than half of respondents were men (52.5%). Moreover, a typical respondent was middle-aged (39.7%), graduated from secondary school (47%) and mostly working as an employee with an unlimited work contract (88.8%) in a normal shift schedule (51.9%). The majority of the respondents (71.2%) worked in non-human serviced fields and the overall proportion of those in human services occupations, i.e., jobs focusing on individuals as clients, made up 29% of the sample. Six per cent (*N* = 22,307) of them worked in the social sector, 5.2% (*N* = 19,621) in healthcare, 8.3% (*N* = 31,463) in education and 9.5% (*N* = 35,993) in other human services areas.

**Table 1:** Sample characteristics.

		***N* (%)**
Sex	Male	199,408 (52.5)
	Female	180,351 (47.5)
Age	<35	91,891 (24.2)
	35–50	150,937 (39.7)
	>50	136,931 (36.1)
Education	Low	61,122 (16.1)
	Medium	178,034 (47.0)
	High	139,921 (36.9)
Country	Austria	10,793 (3.1)
	Belgium	17,484 (5.1)
	Switzerland	7,449 (2.2)
	Cyprus	4,251 (1.3)
	Czech Republic	14,255 (4.1)
	Germany	23,060 (6.8)
	Denmark	12,324 (3.7)
	Estonia	7,210 (2.1)
	Spain	37,275 (10.8)
	Finland	10,439 (3.0)
	Greece	16,296 (4.7)
	Hungary	21,288 (6.2)
	Italy	42,760 (12.4)
	Lithuania	5,323 (1.6)
	Luxemburg	5,089 (1.5)
	Latvia	3,969 (1.2)
	Norway	12,779 (3.7)
	Romania	21,662 (6.4)
	Sweden	11,561 (3.4)
Employment	Permanent job or work contract	18,105 (11.2)
	Permanent job or work contract of unlimited duration	143,185 (88.8)
Atypical work	Never	194,321 (51.9)
	Usually	180,416 (48.1)
Work experience (year)		
Mean (SD)		10.63 (10.34)
Occupation	Social care	22,307 (6.0)
	Healthcare	19,621 (5.2)
	Education	31,463 (8.3)
	Other human services	35,993 (9.5)
	Non-human	270,375 (71.2)

All percentages are based on valid responses.

### 3.2 High prevalence of mental health risk among employees in Europe

**Figure 1:**
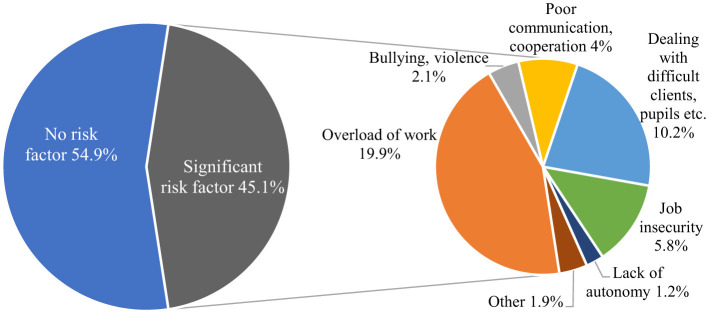
Exposure to work-related risk factors of mental health in 19 European countries. Source: EU-LFS 2020 *ad hoc* module.

Our results showed that 45.1% of employees aged 15–64 (non-student and active) reported at least one workplace factor that negatively affected their mental wellbeing (Figure 1). In other words, nearly one in two workers felt at risk of adverse mental health at work. The most frequently reported risk factors of mental wellbeing were work overload due to time pressure or extended working hours (19.9%), dealing with difficult clients, patients and children (10.2%), and job insecurity (5.8%). To a lesser extent, inadequate communication and cooperation within the organization (4%), bullying and violent behavior (2.1%), as well as lack of autonomy (1.3%) were reported as the prominent risk factor of mental wellbeing among the employees.

We found significant differences in the prevalence of specific mental health risk factors according to sex, age, and educational attainment. Significantly more women than men reported at least one psychosocial risk factor of mental health (Table 2). We found significant sex (47% vs. 43%, *P* = 0,000) differences in the prevalence of specific mental health risk factors (Supplementary Appendix Table S1), i.e., significantly more women reported challenges with difficult clients/patients/children than men (13.1% vs. 8.3%, *P* = 0.000), while significantly more men perceived job insecurity as a prominent mental health risk than women (6.4% vs. 4.9%, *P* = 0.000). We found no significant sex differences in any other mental health risk factors.

**Table 2:** Exposure to mental health risks at work according to sex, age, and educational attainment.

	**Affected by mental health risk at work**		**Total (*N*)**
	**Yes**	**No**	
**Sex (*****p*** = **0.000)**			
Man	43.3	56.7	100.0% (192,593)
Woman	47.0	53.0	100.0% (173,782)
**Age (*****p*** = **0.000)**			
<35	42.3	57.7	100.0% (88,456)
35–50	47.5	52.5	100.0% (146,084)
>50	44.4	55.6	100.0% (131,835)
**Education (*****p*** = **0.000)**			
Elementary	36.2	63.8	100.0% (59,232)
Secondary with diploma	42.6	57.4	100.0% (171,898)
Higher	51.9	48.1	100.0% (134,658)

Source: EU-LFS, 2020 ad hoc module.

Our results demonstrated that respondents aged between 35–50 years reported significantly higher rates of mental health risk compared to younger and older employees (47.5% vs. 42.3% and 44.4%, respectively, *p* = 0.000; Table 2). In the total sample, respondents aged between 35–50 years reported the highest prevalence of work overload and job insecurity, whilst those under the age of 35 perceived difficult clients/patients/children as a prominent mental health risk (Supplementary Appendix Table S1).

Our data also showed that the highest prevalence of mental health risks was reported by respondents with the highest level of educational attainment (Table 2). In contrast, respondents with the lowest educational attainment reported the highest prevalence of mental health risk attributed to job insecurity (Supplementary Appendix Table S1).

### 3.3 Significant differences in exposure to mental health risks across various human services occupations and across different regions in Europe

**Figure 2:**
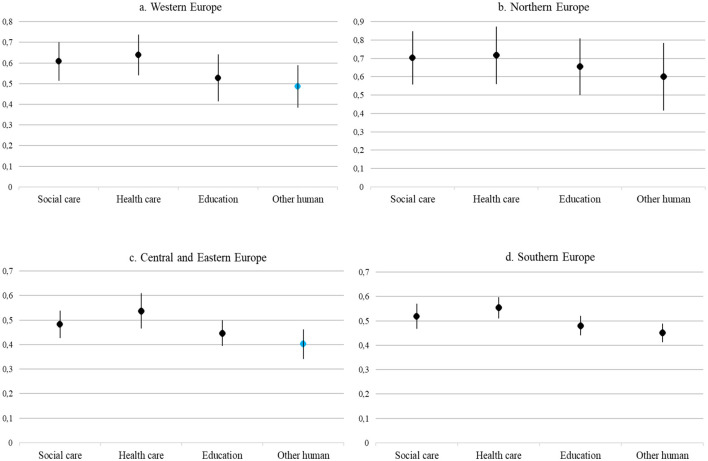
Average Marginal Effects (AME) of human services occupations depicting the likelihood of exposure to mental health risk in the different country groups (Reference Category: non-human services occupations). Black dots indicate effects significant at minimum 0.05 level, while blue dots indicate non-significant effects. Reference category: non-human services occupations. All models controlled for age, sex, education, permanent job, atypical work schedule, work experience, national-level legislation, and PRM index. Source: EU-LFS 2020 *ad hoc* module. **(A)** Western Europe. **(B)** Northern Europe. **(C)** Central and Eastern Europe. **(C)** Southern Europe.

As a next step, we examined the correlations of perceived mental health risks with various human services occupations. Figure 2 illustrates the outcomes of logistic regression analysis. We assessed the correlations for the four groups of countries separately. As parameters *B* of different models cannot be compared in case of logistic regression models, instead of parameters *B*, of the we used—methods developed to compare estimates for different subsamples—Average Marginal Effects (AME) to present and interpret the model results, as described by Mize (33). AME values of the same variable can be compared in different subsamples.

Our results demonstrated that there was a significant correlation between exposure to mental health risk and the type of human services occupations in each group of countries (Figure 2). Our results showed that across Europe, workers in social care, healthcare, education and other human services occupations were more likely to be exposed to psychosocial risk factors of mental health at work than those working in non-human services fields. Of the types of human services occupations, healthcare workers were most likely to be exposed to psychosocial mental health risks at work in all four European regions (AME ranged from 0.537 to 0.717). Employees in the healthcare field in Northern Europe had the highest probability of exposure to mental health risk (AME = 0.717), followed by employees in Western Europe (AME = 0.639), then in Southern Europe (AME = 0.554) and then in Central and Eastern Europe (AME = 0.537). Compared to healthcare, exposure to mental health risks was lower, but also significant in the social care field, with the highest coefficient estimation values observed in Northern Europe (AME = 0.702) and Western Europe (AME = 0.608), respectively. In addition, those involved in education in all four groups of countries (AME values range from 0.447 to 0.655) were more likely to report exposure to mental health risk factors at the workplace compared to those working in non-human services fields. Other human services occupational areas (e.g., human resource management and customer care) were only related to a higher likelihood of mental health risks in Northern and Southern Europe and they do not have as significant an impact as healthcare, social care and education (in Northern Europe AME = 0.600; in Southern Europe AME = 0.451).

### 3.4 Working in various human services occupations predict most prevalent mental health risks

The results of multinomial regressions predicting most prevalent mental health risk factors (i.e., work overload, dealing with difficult clients, and job insecurity) are presented in Tables 3–6.

**Table 3:** Relationships between the most prevalent psychosocial risk factors of mental health at work and the type of human services occupation among employees in Western Europe.

	**Western Europe**							
	***Dependent variable:*** **Most prevalent mental health risk factors**							
	**(Ref: Employees not exposed to mental health risk factors in the workplace)**							
	**Overload of work**		**Dealing with difficult clients, pupils, etc**.		**Job insecurity**		**Other** ^†^	
	**Average marginal effect (SE)**	**95% CI**	**Average marginal effect (SE)**	**95% CI**	**Average marginal effect (SE)**	**95% CI**	**Average marginal effect (SE)**	**95% CI**
**Occupation (Ref: non-human)**								
Social care	0.208^***^ (0.006)	0.196–0.220	0.185^***^ (0.006)	0.173–0.197	0.034^***^ (0.002)	0.029–0.038	0.147^***^ (0.005)	0.137–0.158
Healthcare	0.273^***^ (0.008)	0.257–0.289	0.142^***^ (0.006)	0.130–0.154	0.013^***^ (0.001)	0.009–0.017	0.110^***^ (0.004)	0.101–0.120
Education	0.174^***^ (0.005)	0.164–0.184	0.138^***^ (0.004)	0.129–0.148	−0.024^***^ (0.002)	0.020–0.028	0.103^***^ (0.004)	0.096–0.111
Other human services	0.196 (0.002)	0.185–0.206	0.074 (0.002)	0.068–0.086	0.033 (0.002)	0.028–0.037	0.093 (0.003)	0.086–0.099
**Random-effects parameters**								
Individual-level variance	0.124^***^ (0.008)	0.098–0.147						
Country-level intercept variance	0.282^***^ (0.021)	0.254–0.313						
Log likelihood	−128,791.47							
*N*	100,408							
Number of groups (countries)	5							

The standard errors (SE) are reported in parentheses. In all models, age, sex, education, permanent job, atypical work schedule, work experience, national-level legislation, and PRM index were controlled for.

CI, confidence interval; Ref, reference category.

*** p < 0.001.

† Other risk factors include harassment and violence, lack of autonomy, etc.

Source: EU-LFS 2020 ad hoc module.

**Table 4:** Relationships between the most prevalent psychosocial risk factors of mental health at work and the type of human services occupation among employees in Northern Europe.

	**Northern Europe**							
	***Dependent variable:*** **Most prevalent mental health risk factors**							
	**(Ref: Employees not exposed to mental health risk factors in the workplace)**							
	**Overload of work**		**Dealing with difficult clients, pupils, etc**.		**Job insecurity**		**Other** ^†^	
	**Average marginal effect (SE)**	**95% CI**	**Average marginal effect (SE)**	**95% CI**	**Average marginal effect (SE)**	**95% CI**	**Average marginal effect (SE)**	**95% CI**
**Occupation (Ref: non-human)**								
Social care	0.335^***^ (0.007)	0.320–0.351	0.132^***^ (0.008)	0.117–0.148	−0.039^***^ (0.003)	0.032–0.045	0.233^***^ (0.007)	0.219–0.247
Healthcare	0.381^***^ (0.009)	0.362–0.400	0.136^***^ (0.005)	0.125–0.147	−0.031^***^ (0.003)	0.023–0.038	0.211^***^ (0.008)	0.194–0.228
Education	0.329^***^ (0.007)	0.315–0.343	0.132^***^ (0.007)	0.118–0.146	−0.044^***^ (0.003)	0.038–0.052	0.168^***^ (0.006)	0.155–0.180
Other human services	0.302^***^ (0.008)	0.286–0.319	0.078(0.004)	0.069–0.088	0.084 (0.005)	0.074–0.094	0.152^***^ (0.006)	0.139–0.164
**Random-effects parameters**								
Individual-level variance	0.112^***^ (0.009)	0.097–0.126						
Country-level intercept variance	0.217^***^ (0.014)	0.203–0.238						
Log likelihood	−16,796.44							
*N*	44,507							
Number of groups (countries)	4							

The standard errors (SE) are reported in parentheses. In all models, age, sex, education, permanent job, atypical work schedule, work experience, national-level legislation, and PRM index were controlled for.

CI, confidence interval; Ref, reference category.

*** p < 0.001.

† Other risk factors include harassment and violence, lack of autonomy, etc.

Source: EU-LFS 2020 ad hoc module.

**Table 5:** Relationships between the most prevalent psychosocial risk factors of mental health at work and the type of human services occupation among employees in Central and Eastern Europe.

	**Central and Eastern Europe**							
	***Dependent variable:*** **Most prevalent mental health risk factors**							
	**(Ref: Employees not exposed to mental health risk factors in the workplace)**							
	**Overload of work**		**Dealing with difficult clients, pupils, etc**.		**Job insecurity**		**Other** ^†^	
	**Average marginal effect (SE)**	**95% CI**	**Average marginal effect (SE)**	**95% CI**	**Average marginal effect (SE)**	**95% CI**	**Average marginal effect (SE)**	**95% CI**
**Occupation (Ref: non-human)**								
Social care	0.136^***^ (0.007)	0.128–0.141	0.303^***^ (0.010)	0.283–0.323	−0.037^***^ (0.005)	0.028–0.047	0.078^***^ (0.006)	0.065–0.091
Healthcare	0.154^***^ (0.007)	0.144–0.165	0.238^***^ (0.008)	0.221–0.255	−0.025^***^ (0.004)	0.017–0.034	0.063^**^ (0.005)	0.053–0.073
Education	0.144^***^ (0.005)	0.135–0.156	0.228^***^ (0.007)	0.213–0.243	−0.044^***^ (0.004)	0.031–0.049	0.054 (0.004)	0.046–0.061
Other human services	0.113 (0.006)	0.102–0.126	0.136^***^ (0.005)	0.125–0.147	0.061 (0.004)	0.052–0.069	0.052 (0.004)	0.045–0.059
**Random-effects parameters**								
Individual-level variance	0.107^***^ (0.007)	0.094–0.121						
Country-level intercept variance	0.241^***^ (0.007)	0.232–0.256						
Log likelihood	−25,781.35							
*N*	83,363							
Number of groups (countries)	4							

The standard errors (SE) are reported in parentheses. In all models, age, sex, education, permanent job, atypical work schedule, work experience, national-level legislation, and PRM index were controlled for.

CI, confidence interval; Ref, reference category.

^**^p < 0.01.

*** p < 0.001.

† Other risk factors include harassment and violence, lack of autonomy, etc.

Source: EU-LFS 2020 ad hoc module.

**Table 6:** Relationships between the most prevalent psychosocial risk factors of mental health at work and the type of human services occupation among employees in Southern Europe.

	**Southern Europe**							
	***Dependent variable:*** **Most prevalent mental health risk factors**							
	**(Ref: Employees not exposed to mental health risk factors in the workplace)**							
	**Overload of work**		**Dealing with difficult clients, pupils, etc**.		**Job insecurity**		**Other** ^†^	
	**Average marginal effect (SE)**	**95% CI**	**Average marginal effect (SE)**	**95% CI**	**Average marginal effect (SE)**	**95% CI**	**Average marginal effect (SE)**	**95% CI**
**Occupation (Ref: non-human)**								
Social care	0.246^***^ (0.008)	0.231–0.262	0.142^***^ (0.006)	0.129–0.154	0.080 (0.005)	0.069–0.090	0.090^***^ (0.005)	0.078–0.101
Healthcare	0.190^***^ (0.006)	0.179–0.202	0.149^***^ (0.008)	0.136–0.163	−0.056^**^ (0.005)	0.047–0.065	0.103^**^ (0.006)	0.092–0.114
Education	0.199^***^ (0.005)	0.189–0.209	0.134^***^ (0.005)	0.124–0.144	−0.065^***^ (0.004)	0.057–0.073	0.087^***^ (0.004)	0.079–0.096
Other human services	0.179^**^ (0.002)	0.175–0.183	0.090^**^ (0.004)	0.083–0.097	0.078^**^ (0.003)	0.071–0.084	0.077 (0.003)	0.075–0.088
**Random-effects parameters**								
Individual-level variance	0.116^***^ (0.008)	0.105–0.127						
Country-level intercept variance	0.238^***^ (0.010)	0.226–0.245						
Log likelihood	−52,847.50							
*N*	93,272							
Number of groups (countries)	3							

The standard errors (SE) are reported in parentheses. In all models, age, sex, education, permanent job, atypical work schedule, work experience, national-level legislation, and PRM index were controlled for.

CI, confidence interval; Ref, reference category.

^**^p < 0.01.

*** p < 0.001.

† Other risk factors include harassment and violence, lack of autonomy, etc.

Source: EU-LFS 2020 ad hoc module.

Our results showed that employees working in the healthcare sector in Western, Northern and Central and Eastern Europe were most likely to report work overload as the most prevalent psychosocial risk factor of mental health as evidenced by AME values (0.273, 0.381, and 0.154 in Western, Northern and Central and Eastern Europe, respectively). Among all the regions in Europe, employees working in the healthcare sector in Northern Europe were the most likely to perceive work overload as the most prevalent mental health risk (AME = 0.381). Employees working in other human services (other than healthcare, social care and education) in Central and Eastern Europe were the least likely to report work overload as the most prevalent mental health risk factor (AME = 0.113). We also found that working in the healthcare sector in Central and Eastern Europe was the least likely to increase the risk of mental health (AME = 0.154) attributed to work overload compared to that in all other regions in Europe. Work overload was the most prevalent mental health risk factor among employees working in social care in Southern Europe (AME = 0.246). Compared with employees working in the educational sector in all other European regions, Northern European professionals in the educational sector were most likely to report work overload as the most prevalent mental health risk (AME = 0.329).

Compared to all other regions, employees working in the social care sector in Central and Eastern Europe were most likely to report dealing with difficult clients as the most prevalent mental health risk factor (AME = 0.303). Dealing with difficult clients was the least likely to be reported by employees working in social care and education in Northern Europe as the most prevalent metal health risk (AME = 0.132 in each occupational sector).

Our results demonstrated that employment in various occupational groups in human services had a limited albeit significant impact on the likelihood of reporting job insecurity as the most prevalent mental health risk. Employment in social services (AME = 0.034) and healthcare (AME = 0.013) in Western Europe increased the likelihood of perceiving mental health risks due to job insecurity. A comparison of average marginal effects also showed that the lowest AME values were calculated for employees in healthcare (AME = −0.065) and social care (AME = −0.056) in Southern Europe.

We found that employment in the social care sector in Western, Northern and Central and Eastern Europe predicted the perception of other risk factors (e.g., harassment and violence at work, lack of autonomy at work, inadequate communication and cooperation within the organization) as the most prevalent mental health risk (AME = 0.233 in Northern Europe, AME = 0.147 in Western Europe, AME = 0.078 in Eastern and Central Europe). In Southern Europe, working in the healthcare sector (AME = 0.103) showed a significant correlation with other variables as the most prevalent mental health risk factor. Employment in the educational sector also showed a positive marginal effect on reporting other mental health risk factors most frequently in all four groups of countries, although AMEs were much lower than those observed in the healthcare and social care sectors.

Of the four regions, only employees in Northern Europe working in other human services professions (e.g., customer care professionals) demonstrated a significant AME value (AME = 0.152), suggesting that they were more likely to be affected by health risks due to harassment, violence, lack of autonomy at work, inadequate communication, cooperation within the organization or other factors compared to the employees in non-human services occupations used as a reference.

## 4 Discussion

Our research objective was to identify prominent psychosocial risk factors of mental health in various human services occupations. We also aimed to explore the predictive relationships between employment in different human service sectors and specific mental health risk factors among employees from 19 countries in Europe.

Our results showed that a high proportion of European employees—almost one in two—was at risk of adverse mental health hazards at work. In line with previous research (10, 12, 13, 15), we found that across Europe, professionals in social care, healthcare, education and other human services are proportionally more exposed to psychosocial mental health risks at work than those working in non-human services fields. This can be explained by high emotional stress, frequent traumatic situations (secondary traumatic stress) (34) and intense interpersonal interactions, which can lead to emotional exhaustion and burnout. On the other hand, workplace conditions such as high workloads (35), limited resources and inadequate support (54) further increase their vulnerability to mental health risks. We also found that women, middle-aged employees, and those with higher educational attainment were more at risk, which are also in line with the conclusions of previous research (12). We also demonstrated that the largest proportion of respondents perceived work overload as the most significant psychosocial risk factor of mental health at work, which confirm data from previous research (11). We identified two additional prevalent psychosocial risk factors, i.e., dealing with difficult clients/patients/children and job insecurity. These results are in line with data obtained from previous research by our study group (36) conducted in the social care and educational sectors.

Results also revealed that female sex and younger age significantly correlate with higher rates of reporting difficult interactions with clients/patients/children. This can partly be explained by the fact that the proportion of women in the human services professions is a priori higher (37–40). Human services roles related to care, nursing and support inherently imply more frequent and intimate interaction with clients, which increases the possibility of exposure to health risks arising from conflicted and challenging interactions. The vulnerability of people under 35 is also consistent with previous results. In human services professions, longer work experience proved to be a protective factor (36, 55), since it takes time to learn how to tackle difficult situations associated with clients.

It also turned out that middle-aged employees were most at risk of adverse mental health due to work overload and job insecurity. This can probably be explained, on the one hand, by the career ambitions of 35–50-year-olds and the sacrifices that this entails both at work and at home. On the other hand, today's technological advancements, the option for permanent availability and workplace culture can also play a role in enhancing work overload. The culture of certain workplaces can encourage long working hours and a hustle mentality, not only in the early but also in the middle stages of a career (41).

Our results also revealed that men were more at risk of adverse mental health resulting from frequent perceptions of job insecurity. This may be due, for example, to the fact that, despite the convergence of gender roles, there is still a pay gap between women and men in favor of men (42–47). This pay gap can put financial pressures on men as the primary breadwinner in the household, leading to a more pronounced perception of the risk of losing their job.

Another aim of our study was to explore the correlations between psychosocial risk factors of mental health at work and the type of human services field by means of multilevel logistic and multinomial regression analyses. We confirmed and added to previous research (18, 20–22, 48–50) by identifying the predictive role of specific human services fields in mental health due to unique psychosocial risk factors. Our research showed that working in healthcare and social care were the strongest predictors of reporting work overload and dealing with difficult clients or job insecurity as the most prevalent mental health risk factors.

There may be several potential explanations as to why these human services sectors are the strongest predictors of adverse mental health. People working in both healthcare and social services often encounter needs that require immediate solutions during their work, such as medical emergencies or families/individuals in difficult life situations. In such cases, the demand for services can be particularly intense, which can lead to a high workload. It is also well described that both sectors struggle with labor shortages, so the existing staff have to perform more tasks, which also results in overtime and work overload (35). Many healthcare and social care professionals (e.g., doctors, nurses, emergency responders, social workers) work long and irregular hours, including night shifts and weekends (51). These extraordinary schedules can contribute to physical and mental exhaustion. A special feature of the healthcare and social services sectors, which distinguishes them from the educational and other human services sectors, is, on the one hand, the frequent occurrence of emotionally demanding situations, including the suffering and death of patients (56), or crisis conditions of families and/or individuals. On the other hand, mistakes made in the healthcare and social services professions at work can have very serious consequences. In the long run, both compassion fatigue and the expectation of flawless performance can contribute to work overload, stress, and burnout.

Finally, our research also revealed which Europe countries were most typical of each individual psychosocial risk factor. According to our results, work overload was mainly characteristic of Western, Northern and Central and Eastern European countries and mainly affected healthcare workers. In Southern Europe, mental health risk due to work overload was the highest among social workers. Dealing with difficult clients was the most predominant psychosocial risk factor of mental health in Western and Central and Eastern European countries and affected mainly those working in the social services sector, while in countries in Northern and Southern European it is more likely to impact adversely the mental health of healthcare workers. Looking at Europe as a whole, job insecurity, as a psychosocial risk factor of mental health, had a smaller role than that described above for work overload and dealing with difficult clients: job insecurity was mostly typical of healthcare and social workers in Western Europe. In comparison, in countries in Northern, Central and Eastern Europe, job insecurity represented a greater mental health risk for those working in services of non-human occupational fields. In the social care, healthcare and educational sectors, employees reported job insecurity as the predominant mental health risk to a lesser degree. In other human services occupations (e.g., customer care), other mental health risk factors such as harassment at work, violence, lack of autonomy at work, inadequate communication within the organization, and insufficient cooperation appeared to serve to a small extent as psychosocial mental health risk factors, however, only in Northern Europe.

Although further targeted research is needed for a more in-depth comparison between the countries, our results suggest that the four groups of countries cannot be clearly separated on the basis of the examined psychosocial risk factors of mental health at work. A certain coexistence can be observed between the Northern and Central and Eastern European countries in terms of work overload and job insecurity as a mental health risk, as well as between the Western and Central and Eastern European countries regarding perceived mental health risks related to dealing with difficult clients. There are also similarities in the likelihood of mental health risk due to other risk factors (e.g., harassment and violence at work, lack of autonomy at work, inadequate communication and cooperation within the organization) among Northern, Western and Central and Eastern European countries. Furthermore, these risk factors were most prevalent in the social services sector. However, these tentative claims require further testing. Due to the obvious cultural, economic, social and socio-political differences of countries, there may be very different explanations behind the similarly perceived health risks. In the future, in addition to the two contextual variables used to address differences between countries and groups of countries, i.e., the national-level legislation on psychosocial risks and the psychosocial risk management index, it is necessary to include additional variables.

One of the weaknesses of our research is that we could only use the supplementary module of the 2020 database from the longitudinal survey, thus no temporal comparison could be made. One reason for this is that only the most recent additional survey in 2020 assessed workplace risks of mental health in the most detailed fashion, which was key to our research. Another limitation is that our analyses only cover those 19 countries where the quality of the data on the two key study variables (human services occupations and mental health risk exposure) was adequate.

In addition to the limitations, our research also has several strengths. On the one hand, this is one of the first studies, which includes a comparative analysis of psychosocial risk factors of mental health in various human services sectors among European countries. This international comparison features several dimensions with rich data: spatial, inter-country; between human and non-human services fields; and a dimension comparing specific human services occupations, focusing on social care, healthcare and educational sectors. On the other hand, our study can be linked and can add important evidence to European Agency for Occupational Safety and Health (EU-OSHA)'s research on occupational safety and health in the health and social care sector, which currently in progress. In addition to ergonomic, biological, chemical, and physical risks, the research pays special attention to psychosocial risks. Finally, our evidence-based results draw attention to the specific mental health risk factors of different human services employment areas, which contribute to raising awareness of the topic.

## 5 Conclusion

In conclusion, our research adds evidence to the understanding of the impact of employment in specific human services occupations on the risks to mental health at work. Furthermore, our results on the specific occupational stressors of mental health in each of the human services professions examined support the development of policy and interventional programmes to enhance employees' health and safety and to maintain a high quality of client-centered service.

## Data Availability

The raw data supporting the conclusions of this article will be made available by the authors, without undue reservation.

## Footnotes

^1^Based on advice obtained LFS user guide we deleted the missing cases instead of using imputation methods.
