# Supplementary material for: Mental health risk in human services work across Europe: the predictive role of employment in various sectors
Source: Front Public Health. 2025 Jan 13;12:1407998. doi: 10.3389/fpubh.2024.1407998 (PMC11780093; doi:10.3389/fpubh.2024.1407998)
Supplement: Supplementary file 1 [file Table_1.docx]

**Supplementary Appendix**

**Mental health risk in human services work across Europe: the predictive role of employment in various sectors**

*Ágnes Győri – Éva Perpék – Szilvia Ádám*

**S1***. Description of the occupations in each occupational category*

1) Social care occupations include managers in childcare, care for the elderly and social services, social workers, social counsellors, other social workers (e.g., social politicians), childcare workers, helpers, home care workers and people in personal care professions not elsewhere classified (n.e.c), e.g., care providers working at the patients’ home.

2) Health care occupations include: general practitioners, specialists, graduate and non-graduate nurses, midwives, obstetric assistants, traditional and alternative healers, paramedical professions, dentists, pharmacists, hygienists, physiotherapists, dietitians and nutritionists, audiologists and speech therapists, optometrists, n.e.c. health professionals, medical imaging, diagnostic and therapeutic equipment technicians, medical and pathology lab technicians, pharmaceutical technicians and assistants, medical aids and dental technicians, traditional medicine and alternative medicine professionals and their assistants, dental assistants and therapists, technicians for health records and documentation, community health professionals, opticians, physiotherapy technicians and assistants, medical assistants, environmental and occupational-medicine inspectors, paramedic nurses, social care administrators, medical clerks and health professionals n.e.c. (e.g., semi-skilled nurses, operating room assistants, etc.).

3) Educational and pedagogical occupations includes educational leaders, university and other higher education teachers, vocational trainers, secondary school teachers, primary school teachers, kindergarten teachers, education and methodological occupations, special education teachers, music teachers, language teachers, other teachers, information technology teachers, and educational occupations n.e.c.

4) Other human occupations include the following occupations in law, social sciences, economics or management, clerical and administrative occupations: legal professions, psychologists, religious occupations, human resource management occupations, call center clerks, receptionists, customer information professionals n.e.c., general office occupations (e.g., office administrator, secretary).

**S2. *Table 1.*** The proportion (%) of the most prevalent mental health risk factor at work by gender, age, and educational attainment

|  | No risk factor | Overload of work | Bullying and violence | Poor commu-nication or cooperation | Dealing with difficult clients, patients, pupils etc. | Job insecurity | Lack of autonomy | Another risk factor | *Total* |
| --- | --- | --- | --- | --- | --- | --- | --- | --- | --- |
| *Sex* |  |  |  |  |  |  |  |  |  |
| Man | **56.7** | 20.0 | 1.8 | 3.6 | **8.4** | **6.3** | 1.4 | 1.8 | 100.0% (192,593) |
| Woman | **53.0** | 19.4 | 2.1 | 3.9 | **13.1** | **5.5** | 1.2 | 1.7 | 100.0% (173,782) |
| *Age* |  |  |  |  |  |  |  |  |  |
| <35 | **57.7** | **17.2** | 1.8 | 3.6 | **11.0** | **6.0** | 1.2 | 1.6 | 100.0% (88,456) |
| 35–50 | **52.5** | **21.2** | 2.0 | 3.9 | **10.8** | **6.4** | 1.4 | 1.7 | 100.0% (146,084) |
| >50 | **55.6** | **20.3** | 2.0 | 3.6 | **9.9** | **5.4** | 1.3 | 2.0 | 100.0% (131,835) |
| *Education* | | | | | | | | | |
| Elementary | **63.8** | **14.3** | 1.8 | **3.0** | **6.8** | **7.9** | 1.2 | 1.2 | 100.0% (59,232) |
| Secondary with diploma | **57.4** | **17.3** | 1.9 | **3.5** | **10.6** | **6.3** | 1.2 | 1.7 | 100.0% (171,898) |
| Higher | **48.1** | **25.3** | 2.0 | **4.3** | **12.2** | **4.6** | 1.3 | 2.1 | 100.0% (134,658) |

*Note:* Significant (p<0.05) results are bolded.

*Source: EU-LFS, 2020 ad hoc module*
